# O.1.1-7 As we were and as we should be, concurrent exercise training in adults with schizophrenia: CORTEX-SP study

**DOI:** 10.1093/eurpub/ckad133.083

**Published:** 2023-09-11

**Authors:** Mikel Tous-Espelosin, Nagore Iriarte-Yoller, Pedro Sanchez, Sara Maldonado-Martin

**Affiliations:** GIzartea, Kirola eta Ariketa Fisikoa Ikerkuntza Taldea (GIKAFIT), Society, Sports, and Physical Exercise Research Group, Department of Physical Education and Sport, Faculty of Education and Sport-Physical Activity and Sport Sciences Section, University of the Basque Country (UPV/EHU), Vitoria-Gasteiz, Araba/Álava, Basque Country, Spain; Bioaraba Health Research Institute, Physical Activity, Exercise, and Health Group, Vitoria-Gasteiz, Basque Country, Spain; Bioaraba, New Therapies in Mental Health Group, Vitoria-Gasteiz, Spain; Osakidetza Basque Health Service, Araba Mental Health Network, Psychiatric Hospital of Alava, Vitoria-Gasteiz, Spain; Department of Medicine, Faculty of Health Sciences, University of Deusto, Bilbao, Spain; mikel.tous@ehu.eus; Bioaraba, New Therapies in Mental Health Group, Vitoria-Gasteiz, Spain; Osakidetza Basque Health Service, Araba Mental Health Network, Psychiatric Hospital of Alava, Vitoria-Gasteiz, Spain; Department of Medicine, Faculty of Health Sciences, University of Deusto, Bilbao, Spain; mikel.tous@ehu.eus; GIzartea, Kirola eta Ariketa Fisikoa Ikerkuntza Taldea (GIKAFIT), Society, Sports, and Physical Exercise Research Group, Department of Physical Education and Sport, Faculty of Education and Sport-Physical Activity and Sport Sciences Section, University of the Basque Country (UPV/EHU), Vitoria-Gasteiz, Araba/Álava, Basque Country, Spain; Bioaraba Health Research Institute, Physical Activity, Exercise, and Health Group, Vitoria-Gasteiz, Basque Country, Spain

## Abstract

**Purpose:**

People with schizophrenia (SP) in addition to presenting an unhealthy lifestyle, psychosocial determinants, such as decreased self-esteem and social stigma, and the presence of psychiatric symptoms, have a profound impact on the health-related quality of life (HRQoL). Thus, people with SP have reported greater disability than those healthy control (HC) in HRQoL, assessed by the 36-Item Short-Form Health Survey questionnaire (SF-36). Therefore, the promotion of a healthy lifestyle and interventions aiming to increase physical activity (i.e., aerobic and resistance training) should be a priority given the health benefits. It is well known that regular physical activity improves HRQoL contributing to perceived well-being, with greater improvements in interventions supervised by exercise specialists.

**Methods:**

This research was carried out to analyze the HRQoL measurements in an SP population (n = 112, 20.4% women, 41.3±10.4 yr old) in comparison to an HC sample (n = 30), and to determine in the SP sample the changes in QoL following 20-week supervised concurrent exercise training program (EX) compared to a Treatment-As-Usual (TAU). The SF-36 questionnaire was used to assess HRQoL.

**Results:**

At baseline, the SP population showed lower scores (P < 0.05) in physical function, role-physical, bodily pain, general health, vitality, social functioning, mental health, and physical and mental component summaries compared to HC. After 20-week intervention, physical functioning (↑12.9%), general health (↑15.3%), mental health (↑8.3%), and physical component summary (↑5.1%), increased (P < 0.05) in EX group. In contrast, no significant changes were seen in the TAU for any of the domains studied. Following the Bonferroni correction, there were between-group significant differences. Thus, the EX group showed significant (P < 0.05) improvements (i.e., higher values) compared with the TAU group in physical functioning (difference = 14.3; 95% CI, 5.8-22.8), general health (difference = 11.7; 95% CI, 5.0-18.5) and physical component summary (difference = 4.6; 95% CI, 1.1-8.2), and no significant between-group differences were found in any other domains.

**Conclusions:**

These results highlight the important role of supervised exercise in improving physical and psychological health.

